# Species Composition, Abundance and Seasonal Phenology of Social Wasps (Hymenoptera: Vespidae) in Wisconsin Vineyards

**DOI:** 10.3390/insects9020057

**Published:** 2018-05-31

**Authors:** Christelle Guédot, Katie Hietala-Henschell, Abby N. Lois

**Affiliations:** Department of Entomology, University of Wisconsin-Madison, 1630 Linden Drive, Madison, WI 53706, USA; kghietal@mtu.edu (K.H.-H.); anlois@wisc.edu (A.N.L.)

**Keywords:** *Vespula*, *Dolichovespula*, *Polistes*, acetic acid, isobutanol, heptyl butyrate

## Abstract

Social wasps can be serious pests in fruit growing plantings and are becoming increasingly problematic for grape growers. In this study, we conducted two experiments to assess the species composition and seasonal phenology of social wasps in Wisconsin vineyards in 2015 and 2017. In 2015, three attractants were used: (1) wine; (2) heptyl butyrate (HB); and (3) acetic acid and isobutanol (AAIB) and in 2017, two attractants were used: HB and AAIB. In both years, the same eight species were trapped from the genera *Vespula*, *Dolichovespula*, and *Polistes*. The predominant wasp species trapped were *Vespula maculifrons*, *Vespula vidua*, *Vespula flavopilosa*, and *Vespula germanica* in 2015 and *V. maculifrons*, *V. flavopilosa*, *V. germanica*, and *Dolichovespula maculata* in 2017, in order of total abundance. The populations of *V. vidua* decreased in 2017 compared to 2015, indicating large inter-annual variation. In both years, AAIB lures trapped significantly more *V. flavopilosa*, *V. maculifrons*, and *V. germanica*, the three most prevalent species during grape harvest, than HB, whereas HB lures trapped more *V. vidua* than AAIB. Wine was generally attractive to all species in 2015. This study identifies for the first time the wasp species present in Wisconsin commercial vineyards using chemical attractants. This knowledge, along with the seasonal phenology of these pest species, will help facilitate the development of management strategies for social wasps in commercial vineyards.

## 1. Introduction

Social wasps (Hymenoptera: Vespidae) are important predators of other insects and spiders [1,2,3,4]. However, paper wasps (*Polistes* spp.) and yellowjackets (*Vespula* and *Dolichovespula* spp.) are frequently considered nuisance pests, particularly later in the summer, for people, pets, and livestock [5,1,6,7,8]. Social wasps can become common recurring pests in commercial fruit production, including grape, cherry, peach, pear, apple, and *Myrciaria* sp. [5,9,10,11] and can display aggressive behavior during fruit harvest [12]. Some species, such as *Vespula germanica*, recruit nestmates to desirable food sources [13,14], potentially exacerbating their pest status. In recent years, fruit growers have seen an increase in the numbers of yellowjackets and hornets in orchards, possibly due to softer pesticide programs [15]. Currently, management strategies are limited and include nest treatment or removal, sanitation in the way of damaged and dropped fruit removal, as well as trapping with baits or lures [16,17], but these methods do not provide adequate control in either urban or agricultural settings [18,8].

Eighteen Vespidae species have been identified as damage causing pests to grape worldwide [19]. Social wasps are known to be opportunistic and are often reported as secondary pests utilizing pre-existing holes in grape berries [20]. However, they have also been reported to inflict direct damage to crops, for example in grapes [5,9,21], which may provide the initial entry wound for other pests, such as fungal pathogens or Drosophila species, and may facilitate the transfer of disease-causing microorganisms, such as sour rot in grapes [22]. 

In Wisconsin, yellowjackets and paper wasps are common in urban and rural locations, with the two predominant species being *V. germanica* and *Vespula maculifrons* [23]. Little is known about the diversity and abundance of social wasps occurring in Wisconsin fruit crops, and particularly in vineyards. Identifying the species present in vineyards is fundamental and essential to implement appropriate management strategies for growers as individual wasp species respond differently to chemical attractants and behavioral responses to specific attractants can vary across states and regions [7,23,24,25]. 

Monitoring and management of social wasps have been implemented using different types of semiochemical attractants, reviewed in [18] and various food baits, for example, honey, beef, fruit, yeasts, and fish, e.g., [26,27,28]. Semiochemical attractants are extensively used to monitor and manage pest insects via attract-and-kill, mass trapping, and mating disruption strategies, reviewed in [29] and offer clear advantages over food baits, providing more dependable and standardized attractiveness and at a lower cost over time [18]. 

Currently, no individual bait or lure has been identified to account for all wasp species present in an area and a combination of trapping methods and baits is necessary to assess social wasp species diversity [18,23,30,31]. Several chemical attractants have been identified and are now widely commercialized for managing pestiferous wasps, particularly isobutanol, acetic acid, blends of these two chemicals, and heptyl butyrate, reviewed in [18]. The mixture of acetic acid and isobutanol was shown to be attractive to multiple species, including *Dolichovespula* spp., *Polistes* spp., and *Vespula* spp., in particular species in the *Vespula vulgaris* species group, in different states throughout the USA [23,24,25,31,32]. However, not all *Vespula* spp. are attracted to the acetic acid-isobutanol blend, for example, in Wisconsin *Vespula vidua* was instead attracted to ethyl-(E,Z)-2,4-decadienoate, also known as pear ester [23] and was shown to be attracted to heptyl butyrate, along with other species in the *Vespula rufa* species group, on the East Coast and in Michigan [7,33]. 

This study was designed to (1) assess the species composition of social wasps during the grape growing season; (2) describe the seasonal phenology of the main social wasps present in grape production; and (3) assess the effectiveness of commonly-used attractants on the wasp species present in vineyards. A better understanding of the social wasps present in vineyards and their seasonal flight activity should result in more appropriate recommendations for better management in vineyards.

## 2. Materials and Methods

Two experiments were conducted to assess the species composition and seasonal flight patterns of social wasps in Wisconsin vineyards. All vineyards ranged from 0.5–6 ha (1–15 ac) in size and recently experienced relatively high social wasp populations sometimes associated with crop losses. Vineyards in our study were at least five miles apart and were planted with cold hardy grape cultivars, Marechal Foch, Marquette, Frontenac, St Pepin, and Somerset. Harvest for these cultivars typically occurs from mid-August to mid-September. Social wasps were trapped at six vineyards in south central Wisconsin (Dane and Iowa Counties) from June 2 until all grapes were harvested (22 September 2015). In 2017, three of the same vineyards were used and the trapping season was extended until two consecutive weeks of no wasps caught in traps (1 June 2017–9 November 2017) in order to describe the complete seasonal phenology of social wasps in Wisconsin vineyards. Overall, weather conditions in both years were typical for this region in both temperature and precipitation.

All experiments were conducted using Trappit^®^ dome traps (Great Lakes IPM Inc., Vestaburg, MI, USA). These traps consist of an opaque yellow plastic bottom receptacle that can hold liquid with a 6-cm diameter hole and funnel to allow wasp entry from the bottom center of the trap, and a clear plastic top cover. In both years, all traps contained either 300 mL of water or 300 mL of a liquid attractant, 0.05% boric acid (Fisher Scientific, Santa Clara, CA, USA) to minimize decomposition of the trapped insects, and a couple of drops of unscented dish liquid soap (~0.15 mL) (Seventh Generation, Burlington, VT, USA) to break the surface tension of the liquid. Traps were attached to the trellis in the fruiting area of the grape vines, about 1.5 m above ground. A complete randomized block design was utilized to compare treatments, with traps randomly rotated weekly. Traps were placed at least 12 m apart and one trap per treatment was placed at each vineyard, with one replicate per vineyard.

In 2015, three types of attractants were used in traps: (1) wine; (2) heptyl butyrate (HB); and (3) acetic acid and isobutanol (AAIB), with six replicates per treatment. The wine treatment consisted of red wine (Charles Shaw Merlot, Trader Joes, Madison, WI, USA). Wine was used as a general wasp attractant as it is known to be attractive to *Vespula* and *Polistes* spp. [34,24]. Chemical lures consisted of a 5-mL load of heptyl butyrate (Sigma-Aldrich© LLC., Worldwide) or a 5-mL load of acetic acid (VWR, Worldwide) and isobutanol (Sigma-Aldrich© LLC., Worldwide) in a 1:1 ratio of each chemical placed in the same vial. In both 2015 and 2017, chemicals were dispensed on cotton in 8 mL polypropylene vials (Nalgene Nunc International, Rochester, NY, USA) and volatiles were released from 3 mm holes drilled in the lid to maintain similar release rates in both years [35]. Vials were hung inside the top cover with a wire of about 75 mm in length above the drowning solution and entrance. Lures were replaced monthly. 

In 2017, the same two chemical attractant treatments were utilized (1) heptyl butyrate and (2) acetic acid and isobutanol, with three replicates per treatment. Chemical lures consisted of a 0.1 mL load of heptyl butyrate in a 4-mL polypropylene vial or a 2-mL load of acetic acid and 4 mL load of isobutanol and lures were replaced every two weeks.

In both years, drowning solutions were replaced and samples were collected weekly. Samples were brought back to the laboratory, and preserved in 70% ethanol until identification. Wasps were identified to species under a dissecting microscope using the “Identification Atlas of the Vespidae (Hymenoptera, Aculeata) of the northeastern Nearctic region” [36]. Voucher specimens are held at the Wisconsin Insect Research Collection, at the University of Wisconsin—Madison. 

In 2015, a one-way ANOVA was conducted for each wasp species using Rcmdr [37] to compare the effect of treatment on the mean number of wasps captured per trap for three treatments (AAIB, HB, wine), with the significance level set at *p* < 0.05 and post-hoc Tukey’s tests to separate treatment means. In 2017, a Student’s *t*-test was conducted using Vassarstats [38] to compare the effect of the two treatments on the mean number of wasps captured per trap for the two chemical attractants [38]. Trap catch data was subjected to the Shapiro-Wilk statistic to test for normality. The normality assumption was not met; thus, data for each year was square root-transformed before analyses. The seasonal phenology of the four most common species was graphed for both the 2015 and 2017 field seasons.

## 3. Results

Eight species in the family Vespidae were trapped in south central Wisconsin vineyards in 2015 and 2017. These were *V. vidua* (de Saussure, 1854), *V. maculifrons* (du Buysson, 1905), *V. germanica* (Fabricius, 1793), *Vespula flavopilosa* (Jacobson, 1978), *Dolichovespula maculata* (Linnaeus, 1763), *D. arenaria* (Fabricius, 1775), *P. dominula* (Christ, 1791), and *P. fuscatus* (Fabricius, 1793).

In 2015, *V. maculifrons* and *V. vidua* were the two most commonly caught wasps with a total of 762 (*n* = 288; mean ± SEM: 2.64 ± 0.58 wasps per trap per week) and 632 wasps (*n* = 288; 2.20 ± 0.34), respectively. *Vespula flavopilosa* and *V. germanica* were also trapped with a total of 362 (*n* = 288; 1.25 ± 0.41) and 206 (*n* = 288; 0.72 ± 0.14), respectively. In 2017, *V. maculifrons* was the most abundant species trapped with a total of 2098 wasps (*n* = 132; 16.01 ± 5.16). *Vespula germanica* was next with 508 wasps (*n* = 132; 3.85 ± 1.26) and *V. flavopilosa* with a total of 401 wasps (*n* = 132; 3.04 ± 1.49). The fourth most common species, *D. maculata*, was relatively rare, with a total of 36 wasps captured (*n* = 132; 0.27 ± 0.08). 

**Figure 1:**
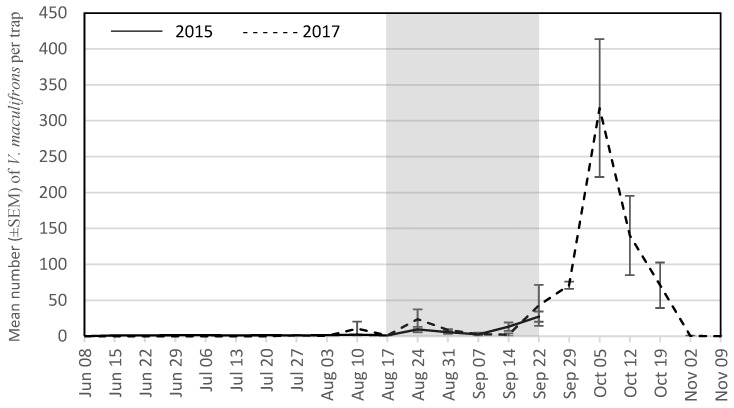
Mean number (±SEM) of *Vespula maculifrons* caught per trap per week in South Central Wisconsin vineyards in traps baited with acetic acid and isobutanol (AAIB) from 8 June to 22 September 2015 (solid line) and from 8 June to 9 November 2017 (dashed line). The shaded region indicates the overall grape harvest for all grape cultivars present across all vineyards sampled.

**Figure 2:**
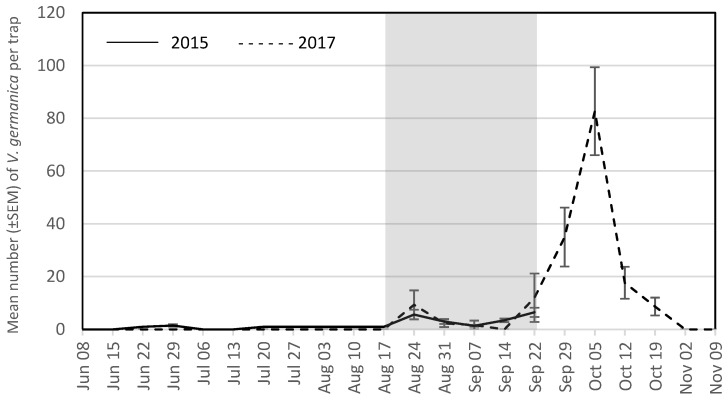
Mean number (±SEM) of *Vespula germanica* caught per trap per week in South Central Wisconsin vineyards in traps baited with acetic acid and isobutanol (AAIB) from 8 June to 22 September 2015 (solid line) and from 8 June to 9 November 2017 (dashed line). The shaded region indicates the overall grape harvest for all grape cultivars present across all vineyards sampled.

**Figure 3:**
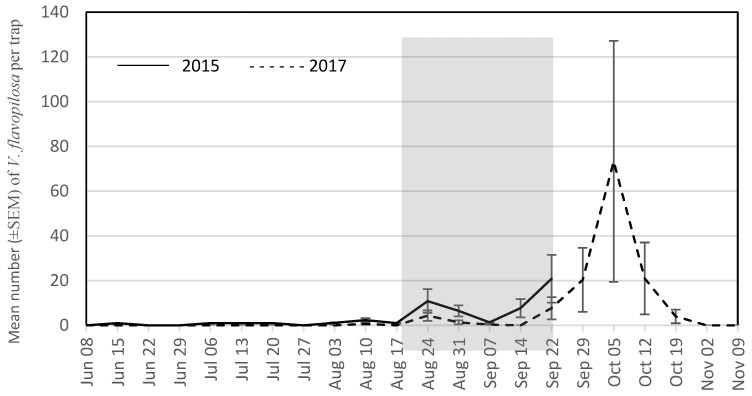
Mean number (±SEM) of *Vespula flavopilosa* caught per trap per week in South Central Wisconsin vineyards in traps baited with acetic acid and isobutanol (AAIB) from 8 June to 22 September 2015 (solid line) and from 8 June to 9 November 2017 (dashed line). The shaded region indicates the overall grape harvest for all grape cultivars present across all vineyards sampled.

**Figure 4:**
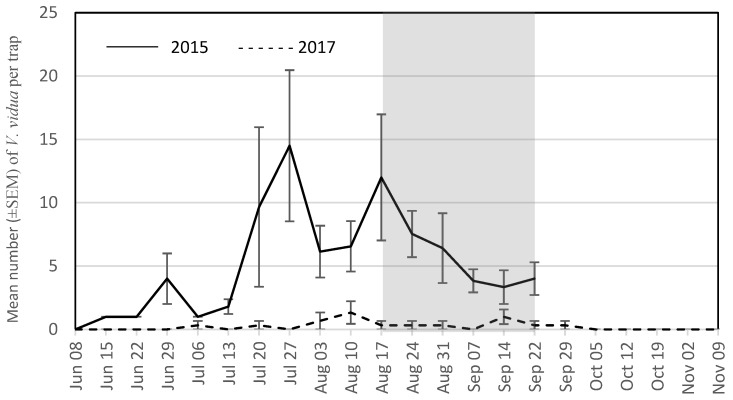
Mean number (±SEM) of *Vespula vidua* caught per trap per week in South Central Wisconsin vineyards in traps baited with heptyl butyrate (HB) from 8 June to 22 September 2015 (solid line) and from 8 June to 9 November 2017 (dashed line). The shaded region indicates the overall grape harvest for all grape cultivars present across all vineyards sampled.

**Figure 5:**
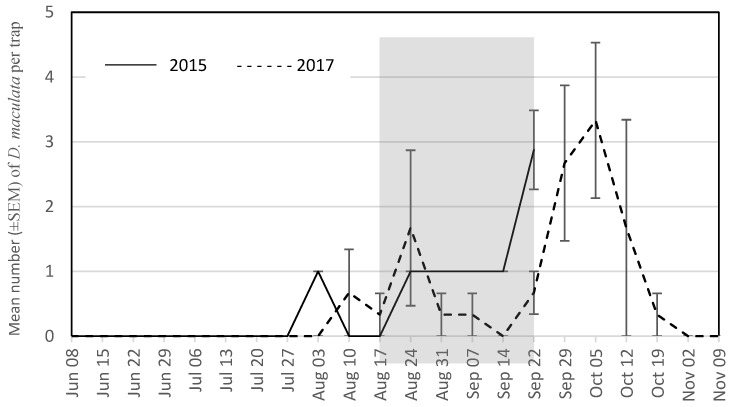
Mean number (±SEM) of *Dolichovespula maculata* caught per trap per week in South Central Wisconsin vineyards in traps baited with acetic acid and isobutanol (AAIB) from 8 June to 22 September 2015 (solid line) and from 8 June to 9 November 2017 (dashed line). The shaded region indicates the overall grape harvest for all grape cultivars present across all vineyards sampled.

The seasonal trap captures are presented for the four most abundant wasp species for each year. During the grape growing season in 2015, these were *V. maculifrons* (Figure 1), *V. germanica* (Figure 2), *V. flavopilosa* (Figure 3), and *V. vidua* (Figure 4). In 2017, the predominant wasp species were *V. maculifrons* (Figure 1), *V. germanica* (Figure 2), *V. flavopilosa* (Figure 3), and *D. maculata* (Figure 5). In 2017, *V. vidua* was very rare compared to 2015 and only 22 wasps were caught throughout the whole trapping season (*n* = 132; 0.17 ± 0.04; Figure 4). The earliest wasps captured by season were *P. fuscatus* and *P. dominula* on 9 June 2015, and *V. vidua* on 6 July 2017. For *V. vidua*, the first wasps were captured in the third week of June in 2015 and populations peaked on July 28 with 14.50 ± 5.97 wasps per trap per week (Figure 4). Populations followed a slow decline and reached 5.33 ± 2.85 wasps per trap per week on the last week of trapping in the third week of September. 

The seasonal flight patterns observed was similar amongst most species in 2017. *Vespula maculifrons* was first detected on 27 July, populations peaked on 5 October (317.67 ± 95.94 wasps per trap per week), and the last individual was trapped on 2 November (Figure 1). *Vespula flavopilosa* was first detected on 10 August, peaked on 5 October (73.33 ± 53.84), and was last caught on 19 October (Figure 3). The first *V. germanica* was trapped on 24 August, populations peaked on 5 October (82.67 ± 16.67), and the last *V. germanica* was caught on 19 October (Figure 2). Similarly, *D. maculata* was first found on 10 August, with peak abundance on 5 October (3.33 ± 1.20), and the last wasp caught on 19 October (Figure 5). The first *V. vidua* was caught on 6 July and numbers increased slightly to a peak of 1.33 ± 0.89 wasps per trap per week, and the last *V. vidua* was captured on 29 September (Figure 4).

In 2015, *V flavopilosa*, *V. vidua*, *V. germanica*, *V. maculifrons*, and *P. fuscatus* were caught in all three treatments (AAIB, HB, wine). Significantly more *V. maculifrons* were caught in traps baited with wine or AAIB than with HB, with no significant difference between wine and AAIB (*F* = 12.3; *df* = 2177; *p* < 0.001; Table 1). More *V. germanica* were trapped with AAIB or wine than with HB (*F* = 8.22; *df* = 2177; *p* < 0.001) and more *V. vidua* were caught with HB than with AAIB or wine, and more with wine than AAIB (*F* = 42.67; *df* = 2177; *p* < 0.001). More *V. flavopilosa* were caught with AAIB and wine than with HB (*F*= 6.97; *df* = 2177; *p* < 0.01), with no significant difference between AAIB and wine. With the two *Dolichovespula* spp., more wasps were caught in the wine than in the HB traps and no significant difference was observed between AAIB and wine or HB (*D. arenaria: F* = 3.77; *df* = 2177; *p* = 0.03; and *D. maculata*: *F* = 6.32; *df* = 2177; *p* < 0.01). Wine caught more *P. fuscatus* (*F* = 20.8; *df* = 2177; *p* < 0.001) and more *P. dominula* (*F* = 4.21; *df* = 2177; *p* = 0.02) than either AAIB or HB.

In 2017, *V. flavopilosa*, *V. vidua*, *V. maculifrons*, and *P. dominula* were caught in both treatments (AAIB and HB). In 2017, more *V. maculifrons* (*t* = 5.18; *df* = 88; *p* < 0.0001), *V. germanica* (*t* = 4.66; *df* = 88; *p* < 0.0001), *V. flavopilosa* (*t* = 3.76; *df* = 88; *p* < 0.0001), *D. arenaria* (*t* = 1.43; *df* = 88; *p* = 0.0001), *D. maculata* (*t* = 4.48; *df* = 88; *p* < 0.0001), *P. fuscatus* (*t* = 3.81; *df* = 88; *p* < 0.0001), and *P. dominula* (*t* = 1.02; *df* = 88; *p* < 0.0001) were caught with AAIB than HB (Table 2). Higher numbers of *V. vidua* were trapped with HB than with AAIB (*t* = 1.86; *df* = 88; *p* = 0.0007; Table 2).

## 4. Discussion

A complex of species from the *Vespula, Dolichovespula*, and *Polistes* genera were present at all the vineyards sampled in both years. The most common wasp species were *V. maculifrons, V. germanica, V. flavopilosa*, and *V. vidua* which was present in relatively high numbers in 2015. *Vespula maculifrons* and *V. germanica* were reported as the most abundant species consistently found in several field trapping experiments in both rural and urban landscapes in Wisconsin [23]. However, the species composition of social wasps can differ between rural and urban landscapes due to differences in life history. For example, *V. germanica* tend to be more commonly found in urban habitats as they build their nests inside structures, whereas *V. maculifrons* prefers rural habitats as it nests underground [39]. Commercial vineyards are usually set in rural areas with several man-made structures and thus may provide the type of nesting habitat suitable for both aerial and underground nesters. To the best of our knowledge, this is the first report documenting the species composition of social wasps in vineyards. 

The composition of species changed slightly from 2015 to 2017, highlighting variation in wasp populations from year to year. Most notably, *V. vidua*, the second most abundant wasp in 2015, was nearly absent in 2017 at the same locations. Fluctuating population dynamics have been documented for *V. germanica* and *V. vulgaris* [40,41,42], likely due to their pest status and propensity for invading new geographical regions [43], and this information is lacking for other species. As noted by Akre and Reed [44], different species exposed to similar weather conditions do not necessarily experience similar fluctuations in population dynamics, which suggests that endogenous biotic factors, such as relative population abundance, may vary by species [42]. More research is necessary to determine if long term population dynamics patterns described for *V. germanica* and *V. vulgaris* apply to other social wasps species, such as *V. vidua*.

In 2017, *V. vidua* was captured, albeit in low numbers, for approximately three months (end of June through September), whereas *V. germanica, V. maculifrons, V. flavopilosa,* and *D. maculata* were trapped for two months or less (mid-August to mid-October). The trapping season for these species coincides with the grape growing season and harvest of the most commonly grown cold hardy grape cultivars in our study. The population abundance of most of the social wasp species found in the study vineyards increases as the grape clusters ripen and become susceptible to damage. Previous reports have shown that social wasps can cause direct damage to sound grape clusters [5,21], which may lead to complete crop loss in vineyards [9]. In addition, foraging wasps have been shown to carry and facilitate the transfer of the sour rot microbial complex, an important disease affecting wine grape quality worldwide [22], exacerbating the pest problem social wasps may pose in vineyards and increasing the need for adequate management strategies.

*Vespula flavopilosa, V. maculifrons*, and *V. germanica* were trapped primarily with the AAIB lures in both years. This result is consistent with previous reports showing that these and other species from the *Vespula vulgaris* group, such as *V. vulgaris* and *V. pensylvanica*, are more attracted to AAIB than HB, reviewed in [18]. Interestingly, it was reported in previous studies that *V. flavopilosa* was either not trapped with AAIB [23] or trapped in low numbers [25], and it was suggested that AAIB is likely not an attractant for *V. flavopilosa* [23]. The results presented herein suggest that *V. flavopilosa* is significantly more attracted to AAIB than HB, and suggest that AAIB is an effective attractant for this species in Wisconsin vineyards.

Species in the *Vespula rufa* group tend to be attracted to HB over AAIB, reviewed in [18] and this is consistent with our findings of catching significantly more *V. vidua* in the HB- than the AAIB-baited traps. We also used wine as a generalist attractant in 2015 and found that wine attracted all species of wasps reported in Wisconsin vineyards, including *P. dominula* which was not caught in any of the AAIB or HB traps. Using different sampling methods, including fruit based baits, was shown to improve the efficiency of surveying social wasps in agricultural settings [30]. Future studies should address other chemical attractants and combinations of these to determine optimal attractants for the predominant wasp species present in vineyards, particularly during grape harvest.

In this study, we reported all social wasps trapped in our experiments. We caught *V. flavopilosa* and very few *P. dominula* which were not trapped in a previous study done in Wisconsin [23], and *P. dominula* and *V. germanica* were not reported in traps baited with either AAIB or HB in Michigan [7]. Species reported in the Midwest and that were not trapped with AAIB, HB, or the general attractant wine in this study, include *V. vulgaris*, *V. acadica,* and *V. consobrina* [7], probably because of the different habitats sampled, that is, vineyards herein and forest in the Michigan study by Reed and Landolt [7]. It remains to be determined if the low numbers of wasps caught for some species, such as *P. fuscatus* or *P. dominula,* is due to a weak response to the chemical attractants placed in traps, low population levels, or low activity of these species in the vineyards sampled [7].

The results provided herein provide a better understanding of the social wasp species assemblages in Wisconsin vineyards and describes the seasonal phenology of the predominant species. These results will help improve management strategies in vineyards to target specific wasp pest species. Indeed, the implementation of attractant traps for monitoring and mass trapping can be targeted to the specific species identified in vineyards using the most appropriate attractant, as species are known to vary in their responses to specific attractants [18].

## Figures and Tables

**Table 1:** Mean ± SEM number of social wasps captured per trap per week baited with acetic acid and isobutanol (AAIB), heptyl butyrate (HB), or wine from 21 July through 22 September 2015 in South Central Wisconsin vineyards.

Wasp Species	AAIB	HB	Wine
*Vespula maculifrons*	4.68 ± 1.82 b	0.32 ± 0.10 a	7.55 ± 1.96 b
*Vespula germanica*	1.50 ± 0.53 b	0.18 ± 0.07 a	1.68 ± 0.35 b
*Vespula vidua*	0.25 ± 0.09 a	7.52 ± 1.24 c	2.38 ± 0.70 b
*Vespula flavopilosa*	3.12 ± 1.67 b	0.03 ± 0.02 a	2.83 ± 0.94 b
*Dolichovespula arenaria*	0.02 ± 0.02 ab	0 ± 0 a	0.10 ± 0.05 b
*Dolichovespula maculata*	0.17 ± 0.10 ab	0 ± 0 a	0.35 ± 0.12 b
*Polistes fuscatus*	0.20 ± 0.06 a	0 ± 0 a	0.75 ± 0.15 b
*Polistes dominula*	0 ± 0 a	0 ± 0 a	0.07 ± 0.03 b

Means within a row followed by the same letter are not significantly different by Tukey’s Test (*p* < 0.05).

**Table 2:** Mean ± SEM number of social wasps captured per trap baited with acetic acid and isobutanol (AAIB) or heptyl butyrate (HB) from 20 July through 2 November 2017 in South Central Wisconsin vineyards.

Wasp Species	AAIB	HB
*Vespula maculifrons*	46.24 ± 14.03 a	0.39 ± 0.25 b
*Vespula germanica*	11.29 ± 3.46 a	0.00 ± 0.00 b
*Vespula vidua*	0.13 ± 0.07 a	0.33 ± 0.10 b
*Vespula flavopilosa*	8.87 ± 4.26 a	0.04 ± 0.03 b
*Dolichovespula arenaria*	0.05 ± 0.03 a	0.00 ± 0.00 b
*Dolichovespula maculata*	0.80 ± 0.22 a	0.00 ± 0.00 b
*Polistes fuscatus*	0.42 ± 0.13 a	0.00 ± 0.00 b
*Polistes dominula*	0.07 ± 0.04 a	0.02 ± 0.02 b

Means within a row followed by the same letter are not significantly different (*p* < 0.05).
